# Efficacy of Low-Dose Paroxetine for the Treatment of Hot Flushes in Surgical and Physiological Postmenopausal Women: Systematic Review and Meta-Analysis of Randomized Trials

**DOI:** 10.3390/medicina55090554

**Published:** 2019-08-31

**Authors:** Gaetano Riemma, Antonio Schiattarella, Marco La Verde, Giuseppina Zarobbi, Simone Garzon, Gaspare Cucinella, Gloria Calagna, Domenico Labriola, Pasquale De Franciscis

**Affiliations:** 1 Department of Women, Child, and General and Specialized Surgery, University of Campania “Luigi Vanvitelli”, 80138 Naples, Italy; 2 Department of Obstetrics and Gynecology, “Filippo Del Ponte” Hospital, University of Insubria, 21100 Varese, Italy; 3 Department of Obstetrics and Gynaecology, University of Palermo, 90133 Palermo, Italy; 4 Obstetrics and Gynecology Unit, “Villa Sofia Cervello Hospital”, University of Palermo, 90133 Palermo, Italy

**Keywords:** paroxetine, hot flushes, menopause, vasomotor symptoms, efficacy, sleep disturbances

## Abstract

*Background and Objectives:* Hot flushes and sleep disturbances are the most common vasomotor symptoms (VMS) reported by postmenopausal women. Hormonal treatment is to date referred to as the gold standard approach but not suitable for all the patients. Alternative treatments are needed in case of a contraindication to menopausal hormone therapy (MHT), adverse side effects, and poor compliance. Paroxetine salt is the only nonhormonal medication approved by the US Food and Drug Administration for the management of VMS. Nonetheless, few trials with low consensus are available about this topic. In this review, we aimed to evaluate the efficacy of low-dose paroxetine therapy in the treatment of vasomotor hot flushes and night sleep disturbances in postmenopausal women. *Materials and Methods:* We performed an electronic search from the beginning of all databases to July 2019. All results were then limited to a randomized trial. Restrictions for language or geographic location were not utilized. Inclusion criteria were randomized clinical trials of physiological or surgical postmenopausal women experiencing hot flushes and sleep disturbances who were randomized to either low-dose paroxetine or placebo (i.e., formulations without active ingredients). The primary outcome evaluated was the mean weekly reduction of hot flushes. *Results:* Five randomized clinical trials, including 1482 postmenopausal women, were analyzed. Significant heterogeneity (I^2^ = 90%) between studies was noted. Hot flushes episodes were significantly reduced in the treatment arm compared to placebo (mean difference (MD) −7.97 [−10.51, −5.92] episodes/week). Results on the improvement on sleep were limited by being reported in only two studies; however, no significant reduction of night-time awakenings was observed (MD, −0.40 awakenings/night [−1.38, 0.58 CI]). *Conclusions:* Low-dose paroxetine is an effective treatment for vasomotor menopause symptoms, including hot flushes.

## 1. Introduction

Menopausal age has a major impact on women’s life. Progressive reduction of ovarian production of estrogens and progesterone develop several symptoms [1,2]. Although changing in intensity and frequency from a woman to another, those symptoms involve vasomotor symptoms (VMS) [3], sleep disturbances, genitourinary syndrome of menopause (i.e., vulvovaginal atrophy) [4,5], and psychologic and emotional disorders [6]. In case of surgical menopause, in which women undergo bilateral oophorectomy before normal menopause, those symptoms are increased compared with the general population [2].

The most common reason for complaint in women is VMS [7], VMS can be typically defined as the presence of daytime hot flushes and “night-sweats” due to hormonal change in menopause, especially hot flushes (or flashes in the USA) and night sweats, which profoundly affect women’s quality of life and quality of sleep [3,6].

For those women, menopausal hormone therapy (MHT) is recognized as the first-line therapy [8]; however, alternative treatments are needed in case of a contraindication to MHT, adverse side effects, and poor compliance [9]. Besides, there are also women who refuse hormonal treatment for a variety of reasons, mainly due to their fear of increasing the risk of cancer or weight gain [10,11]. For this reason, it is necessary to search for nonhormonal treatments from which women could benefit from.

Paroxetine is an antidepressant drug which belongs to the SSRI (selective serotonin reuptake inhibitors) class. At high dose, it is used to treat major depressive disorders [12]. Its efficacy and safety have been proved by several trials and reviews [13]. SSRIs like citalopram or escitalopram were the first to be described effective in reducing VMS severity [14]. In 2013, the United States Food and Drug Administration (US FDA) approved the usage of low-dose paroxetine for the management of hot flushes and night sweats after the end of two phase III randomized trials. Low-dose paroxetine consists of a 7.5 mg daily dose of paroxetine mesylate or a 12.5 mg single dose of paroxetine hydrochloride [15]. Since 2013, studies have been carried out to validate the efficacy of paroxetine on hot flushes and sleep disturbances with different results [14,15,16,17,18,19,20,21,22]. For this reason, the study aim was to carry out a systematic review and meta-analysis of randomized clinical trials in order to evaluate the effectiveness of low-dose paroxetine on hot flushes reduction and its impact on night-time awakenings.

## 2. Materials and Methods

### 2.1. Search Strategy

We performed electronic research in Scopus, ClinicalTrials.gov, MEDLINE, EMBASE, the PROSPERO International Prospective Register of Systematic Reviews, and the Cochrane Central Register of Controlled Trials with the use of the following keywords: “paroxetine,” “hot flushes,” “vasomotor symptoms,” “sleep,” and “menopause” from inception of each database to April 2019. All results were then limited to “clinical trial.” Restrictions for language or geographic location were not applied. Commentaries, editorials, letters, and abstracts were excluded from search in every database. When needed, we obtained unpublished data directly contacting authors of the original papers whenever methodology indicated that further outcome data were recorded.

### 2.2. Study Selection and Risk of Bias

Inclusion criteria were randomized clinical trials of symptomatic women with VMS in physiological or surgical menopause who were experiencing hot flushes and sleep disturbances and were randomized to low-dose paroxetine treatment. Exclusion criteria included quasi-randomized trials and trials in women who were eligible for paroxetine but did not experience sleep disturbances, trial involving high-dose paroxetine administration or other therapy regimens, trials involving premenopausal women, and studies on women suffering from major depressive symptoms before menopause. We piloted the abstraction forms, designed specifically for this review, starting on a sample of included articles. Key characteristics abstracted included patient descriptors, setting, features of the treatment and comparator, outcomes evaluation, study duration, mean follow-up, results, and quality elements.

Two authors (A.S., M.L.V.) reviewed and classified all the abstracts independently. Agreement regarding potential relevance was reached by consensus; the same two authors obtained full-text copies of those papers and independently extracted relevant data about the study characteristics and the reproductive outcomes. When found, all the inconsistencies were discussed by the reviewers and consensus was reached by discussion with a third author (P.D.F.). In addition, authors were contacted whenever information was not reported but the methodology indicated that such information would have been recorded.

The meta-analysis was referred to follow the Preferred Reporting Item for Systematic Reviews and Meta-analyses (PRISMA) statement [16]. The risk of bias in each of the included study was assessed by means of the criteria outlined in the Cochrane Handbook for Systematic Reviews of Interventions [22]. Review authors’ judgments were categorized as “low risk,” “high risk,” or “unclear risk” of bias.

### 2.3. Outcomes

An intention-to-treat approach was used for every part of the analysis; women were evaluated in reference to the treatment group to which they were randomly allocated in the original trials. The primary outcome was the reduction of hot flushes episodes as achieved as a mean weekly reduction (mean and SD). Secondary outcomes were sleep interference reduction, calculated as mean nighttime awakenings reduction (mean and SD), and adverse treatment effects (as achieved as number and percentage from each study). We also carried out a subgroup analysis for the primary outcome between women with surgical menopause and physiological menopause.

### 2.4. Data Analysis

The data analysis was carried out using Review Manager 5.3 (The Nordic Cochrane Centre 2014, Copenhagen, Denmark). The summary measures were reported as summary mean difference (MD) with 95% of confidence interval (CI) using the random-effects model of Der Simonian and Laird. I-squared (Higgins I^2^) greater than 0% was used to identify heterogeneity. P-value < 0.05 was considered statistically significant.

## 3. Results

Initially, we were able to identify nine trials from full-text articles evaluating paroxetine as a treatment for hot flushes in menopause [15,17,18,19,20,21,22,23,24]. Five studies were excluded because they were nonrandomized or cross-over trials [17,18,20,21,22]. Four randomized clinical trials, that included 1482 women who were eligible using inclusion criteria for this meta-analysis, were analyzed by the authors.

**Figure 1:**
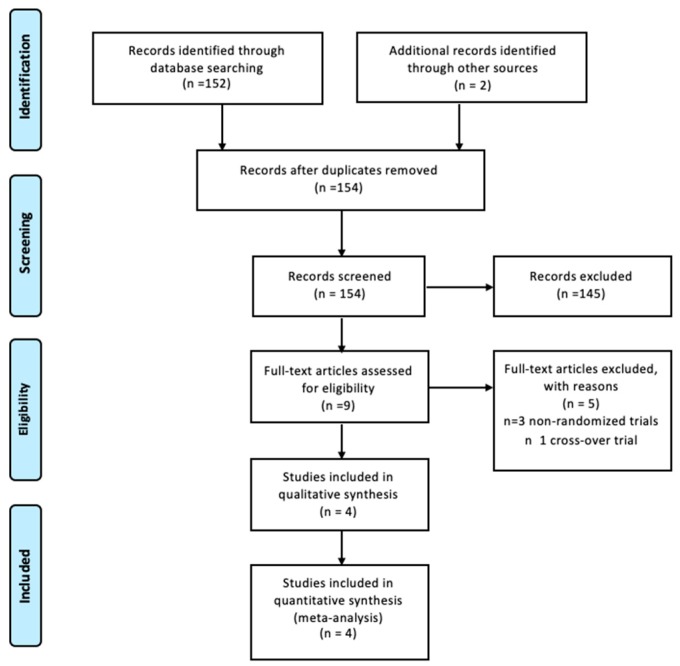
Flow diagram of the studies identified in the systematic review.

Figure 1 shows the flow diagram (PRISMA template) of information through the different phases of the review.

**Figure 2:**
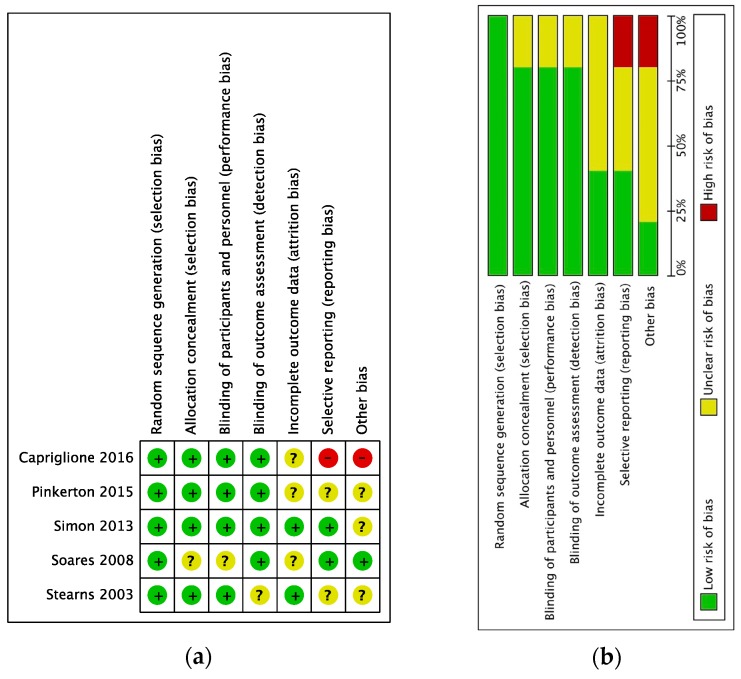
Assessment of risk of bias. (**a**) Summary of risk of bias for every trial; plus sign: low risk of bias; minus sign: high risk of bias; question mark: unclear risk of bias. (**b**) Risk of bias graph about each risk of bias item presented as percentages across all included studies.

The overall risk of bias was judged as low. Most studies had granted a low risk of bias in selective reporting and incomplete outcome data according to the Cochrane Collaboration’s tool. Four of the five studies were double-blind randomized, leading to a low bias judgment (Figure 2). Statistically, heterogeneity within the trials ranged from low to high with no inconsistency (I_2_ = 0%) for the secondary outcomes and I^2^ = 90% for the primary outcome.

Main baseline characteristics of the five included trials are described in Table 1. A total of 1482 women were included in quantitative analysis, 738 were randomized to the treatment (low-dose paroxetine) group and 744 to the placebo group. All studies enrolled women with diurnal and nocturnal hot flushes due to a postmenopausal hormonal status and related sleep disturbances as referred to night awakenings. Four studies involved women with surgical or physiological menopause, Capriglione et al. [21] included only women with a prior history of surgically treated gynecological cancer and subsequent surgical menopause. Women who were randomized into the treatment group received one daily administration of low-dose paroxetine (7.5 or 12.5 mg) for a minimum of 6 to a maximum of 16 months; meanwhile, women into the placebo group received formulations without active ingredient for the same time frame.

### 3.1. Synthesis of Results

**Figure 3:**
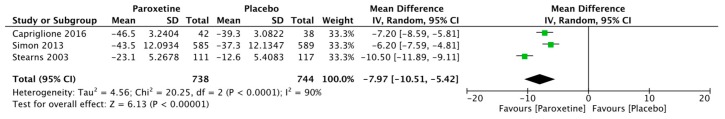
Forest plot for the reduction of hot flushes episodes.

Women who were treated with low-dose paroxetine had a significant reduction of hot flushes episodes (evaluated as mean weekly reduction from baseline) compared to placebo (mean difference −7.97 [−10.51, −5.42] episodes/week) (Figure 3) (Table 2).

In Table 3 subgroup analysis is described, achieved reduction was −7.89 MD [−11.23, −4.81 CI] for patients with physiological menopause and −7.63 MD [−10.15, −5.56 CI] for women with surgical menopause.

**Figure 4:**
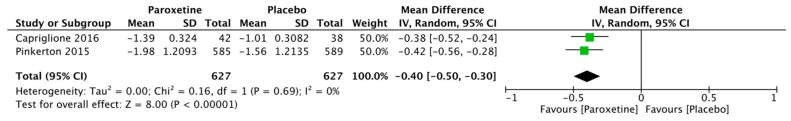
Forest plot for the reduction of nighttime awakenings.

Two randomized clinical trials evaluated the effects on nighttime awakenings. However, there were no statistically significant differences about mean nighttime awakenings reduction between paroxetine and placebo were evaluated (−0.40 episodes/night MD [−1.38, 0.58 CI]) (Figure 4).

### 3.2. Heterogeneity

Together with a small number of studies, however, this systematic review also highlights the significant heterogeneity present in the available studies. This might be related to the fact that population characteristics were variable, as well as which was suboptimal in some of them. In addition, variability was present in other characteristics of the eligible studies, such as the criteria used to describe VMS, the definition of the outcome measures, and the mean follow-up period. It is important to take into account this heterogeneity when interpreting the results of this meta-analysis. For this reason, available data were analyzed with the use of a random-effects model, which uses a conservative approach by acknowledging that the samples evaluated in the individual studies might not all originate from the same population. At the same time, the subgroup analyses conducted in this meta-analysis (surgical menopause vs. physiological menopause) might give the reader some insight about the potential moderating effect of some of these characteristics on the observed effect sizes. Sensitivity analysis on daily hot flushes reduction was conducted. Removing each study one by one reported the analysis to be nonsignificant (*p* > 0.05). However, when Stearns et al. 2013 was removed from the pool, heterogeneity (I^2^) fell from 90 to 0%. This may be related to a different paroxetine dosage (12.5 mg/day) if compared to the other studies since no other substantial differences between populations were found.

## 4. Discussion

This meta-analysis of randomized trials evaluating (physiological or surgical) postmenopausal women suffering from daily hot flushes and sleep disturbances showed that low-dosage paroxetine is an effective treatment for the reduction of daily hot flushes; nonetheless, we did not find paroxetine usage clinically significant in improving sleep parameters such as nighttime awakenings. Furthermore, paroxetine was effective in reducing hot flushes both in women with physiological and surgical menopause.

Simon et al. [19] reported that 50.3% of women reported at least one case of treatment-emergent sleep-related adverse events (TEAEs) from low to moderate (i.e., insomnia, hypersomnia, incubus, reduced sleep length). Stearns et al. [19] also reported that 10% of women experienced light headache during treatment, although it was nonsignificant. Moreover, sporadic cases of dizziness and nausea were found. Concerning adverse effects, trials included in this quantitative analysis did not found any statistically significant differences between the two arms; women under paroxetine or under placebo experienced same adverse effects at similar frequencies [17,18,19,20,21]. Although nausea or dizziness or hypersomnia have been found clearly as main adverse events in reviews that involved paroxetine for major depressive syndromes, we did not find this evidence in our review. This observation could be related to the low-dosage therapy regimen.

However, women who experience breast cancer or thromboembolic venous pathologies and, therefore, not suitable for menopausal hormone therapy can benefit from the use of low-dose paroxetine [22,23,24]. For this reason, the North American Menopause Society described paroxetine as a first-choice drug for those non-suitable patients and for those who refuse hormonal therapy [25]. Together with VMS, paroxetine has been reported to be useful also in decreasing chronic pelvic pain in pre, peri-, and postmenopausal women, leading to an improvement of social relationships, sexuality, and mental health [26,27,28,29,30,31,32]

Other reviews also described and supported the usage of paroxetine for the reduction of hot flushes, leading to similar conclusions [31,32]. However, the impact on sleep disturbances was not considered. Nonetheless, they used to describe VMS deriving them from several different scales instead of considering the full number of episodes and the mean weekly reduction. We believe that this kind of approach is the clearest evidence of the efficacy of paroxetine against hot flashes. Furthermore, no review considered a subgroup analysis dividing women with a surgical from physiological menopause. For those reasons, we believed that an updated meta-analysis of RCTs was needed.

A strength of our study is that it involved only randomized double-blind trials. No open trial or prospective study was considered during study selection. Although heterogeneity has been found in the primary outcome, it is clear that confidence intervals detected overlap, leading to a good quality of evidence.

Severe limitations of our meta-analysis can be found: studies involved in this review did not state the difference between night and daytime VMS; also, the length of each VMS was not evaluated. Furthermore, the low number of RCTs included, high heterogeneity, and the fact that the outcomes of interest are not fully reported in each study may limit the results of this meta-analysis. Moreover, low-dose paroxetine regimen used between studies ranged from 7.5 to 12 mg, for this reason, a random-effect model was used in order to calculate MD and CI; more RCTs involving differences between those two regimens are needed. Furthermore, it was not possible to evaluate publication bias using a funnel plot since the number of studies involved was inferior to 10. Nonetheless, it is also needed to highlight that three [17,19,23] of four studies of this meta-analysis were founded by pharmaceutical companies.

## 5. Conclusions

Low-dosage paroxetine can be useful in the reduction of vasomotor symptoms such as hot flushes in physiological or surgical postmenopausal women. In two RCTs, no improvement of sleep is observed for those women, although the description of sleep-related outcomes is often shifted by the subjective opinions of the patient. Additional studies are required to assess the efficacy of paroxetine on sleep disturbance.

## Figures and Tables

**Table 1:** Characteristics of the included studies.

	Stearns 2003 [17]	Simon 2013 [19]; Pinkerton 2014 [23]	Capriglione 2016 [21]
**Location**	USA	USA	Italy
**Sample size n. (Treatment/Placebo)**	228 (111/117)	1174 (585/589)	80(42/38)
**Treatment administered**	Paroxetine 12.5 mg/day for 6 weeks	Paroxetine 7.5 mg/day for 12 weeks	Paroxetine 7.5 mg/day for 16 weeks
**Inclusion criteria**	Physiologic postmenopausal women with hot flushes and sleep disturbances	Physiologic or surgical postmenopausal women with hot flushes and sleep disturbances	Surgical postmenopausal women with hot flushes and sleep disturbances
**Mean Age (SD): Treatment Placebo**	56.3	54.6 (5.73)	53.5 (5.71)
	56.3	54.5 (6.01)	53.6 (5.01)
**Mean BMI (SD): Treatment Placebo**	Not available	28.62 (5.73)	26.7 (4.62)
		29.03 (5.51)	27.5 (4.71)
**Follow-up**	6 weeks	12 weeks	16 weeks
**Outcomes**	Hot flushes reduction; sleep disturbance	Hot flushes reduction; sleep disturbance	Hot flushes reduction; sleep disturbance

Abbreviations: SD: standard derivation; BMI: body mass index.

**Table 2:** Primary outcomes of included studies.

+		Stearns 2003 [17]	Simon 2013 [19]	Pinkerton 2014 [23]	Capriglione 2016 [21]	I^2^	Mean Difference (MD) and 95% CI
**Hot flushes reduction (mean weekly reduction)**	Paroxetine	−23.1 ± 5.3	−43.5 ± 12.1	Not available	−46.5 ± 3.2	93%	−7.97 [−10.51, −5.42]
	Placebo	−12.6 ± 5.4	−37.3 ± 12.1		−39.3 ± 3.1		
**Sleep interference reduction (mean nighttime awakenings reduction)**	Paroxetine	Not applicable	Not available	−1.98 ± 1.21	−1.39 ± 0.32	0%	−0.40 [−1.38, 0.58]
	Placebo			−1.56 ± 1.21	−1.01 ± 0.31		

**Table 3:** Subgroup analysis for included studies.

Population	Outcome	Included Studies	Total	I^2^	MD (95% CI)
**Physiological postmenopausal**	Hot flushes reduction	Simon 2013, Stearns 2003	615/701 (87.7%) vs. 641/720 (89.0%)	65%	−7.89 [−11.23, −4.81]
**Surgical postmenopausal**	Hot flushes reduction	Capriglione 2016, Simon 2013,	110/766 (14.3%) vs. 100/760 (13.1%)	55%	−7.63 [−10.15; −5.56]

Abbreviations: MD: mean difference; CI: confidence interval.

## References

[1] Nelson H.D. (2008). Menopause. Lancet.

[2] Santoro N., Epperson C.N., Mathews S.B. (2015). Menopausal Symptoms and Their Management. Endocrinol. Metab. Clin..

[3] Kim M.J., Yim G., Park H.Y. (2018). Vasomotor and physical menopausal symptoms are associated with sleep quality. PLoS ONE.

[4] Laganà A.S., Vitale S.G., Stojanovska L., Lambrinoudaki I., Apostolopoulos V., Chiofalo B. (2018). Preliminary results of a single-arm pilot study to assess the safety and efficacy of visnadine, prenylflavonoids and bovine colostrum in postmenopausal sexually active women affected by vulvovaginal atrophy. Maturitas.

[5] Torella M., Del Deo F., Grimaldi A., Iervolino S.A., Pezzella M., Tammaro C., Gallo P., Rappa C., De Franciscis P., Colacurci N. (2016). Efficacy of an orally administered combination of hyaluronic acid, chondroitin sulfate, curcumin and quercetin for the prevention of recurrent urinary tract infections in postmenopausal women. Eur. J. Obstet. Gynecol. Reprod. Biol..

[6] Shaver J.L., Woods N.F. (2015). Sleep and menopause: A narrative review. Menopause.

[7] Deecher D.C., Dorries K. (2007). Understanding the pathophysiology of vasomotor symptoms (hot flushes and night sweats) that occur in perimenopause, menopause, and postmenopause life stages. Arch. Womens Ment. Health.

[8] Krause M.S., Nakajima S.T. (2015). Hormonal and nonhormonal treatment of vasomotor symptoms. Obstet. Gynecol. Clin..

[9] Chang H.Y., Jotwani A.C., Lai Y.H., Jensen M.P., Syrjala K.L., Fann J.R., Gralow J. (2016). Hot flashes in breast cancer survivors: Frequency, severity and impact. Breast.

[10] De Franciscis P., Cobellis L., Fornaro F., Sepe E., Torella M., Colacurci N. (2007). Low-dose hormone therapy in the perimenopause. Int. J. Gynaecol. Obstet..

[11] Laganà A.S., La Rosa V.L., Rapisarda A.M.C., Valenti G., Sapia F., Chiofalo B., Rossetti D., Ban Frangež H., Vrtačnik Bokal E., Vitale S.G. (2017). Anxiety and depression in patients with endometriosis: Impact and management challenges. Int. J. Womens Health.

[12] Purgato M., Papola D., Gastaldon C., Trespidi C., Magni L.R., Rizzo C., Furukawa T.A., Watanabe N., Cipriani A., Barbui C. (2014). Paroxetine versus other anti-depressive agents for depression. Cochrane Database Syst. Rev..

[13] Bourin M., Chue P., Guillon Y. (2001). Paroxetine: A review. CNS Drug Rev..

[14] Stubbs C., Mattingly L., Crawford S.A., Wickersham E.A., Brockhaus J.L., McCarthy L.H. (2017). Do SSRIs and SNRIs reduce the frequency and/or severity of hot flashes in menopausal women. J. Okla. State Med. Assoc..

[15] Simon J.A., Chandler J., Gottesdiener K., Lazarus N., He W., Rosenberg E., Wagner J.A., Denker A.E. (2014). Diary of hot flashes reported upon occurrence: Results of a randomized double-blind study of raloxifene, placebo, and paroxetine. Menopause.

[16] Moher D., Liberati A., Tetzlaff J., Altman D.G., Group P. (2009). Preferred reporting items for systematic reviews and meta-analyses: The PRISMA statement. J. Clin. Epidemiol..

[17] Stearns V., Beebe K.L., Iyengar M., Dube E. (2003). Paroxetine controlled release in the treatment of menopausal hot flashes: A randomized controlled trial. JAMA.

[18] Soares C.N., Joffe H., Viguera A.C., Petrillo L., Rydzewski M., Yehezkel R., Somley B., Cohen L.S. (2008). Paroxetine versus placebo for women in midlife after hormone therapy discontinuation. Am. J. Med..

[19] Simon J.A., Portman D.J., Kaunitz A.M., Mekonnen H., Kazempour K., Bhaskar S., Lippman J. (2013). Low-dose paroxetine 7.5 mg for menopausal vasomotor symptoms: Two randomized controlled trials. Menopause.

[20] Portman D.J., Kaunitz A.M., Kazempour K., Mekonnen H., Bhaskar S., Lippman J. (2014). Effects of low-dose paroxetine 7.5 mg on weight and sexual function during treatment of vasomotor symptoms associated with menopause. Menopause.

[21] Capriglione S., Plotti F., Montera R., Luvero D., Lopez S., Scaletta G., Aloisi A., Serra G.B., Angioli R. (2016). Role of paroxetine in the management of hot flashes in gynecological cancer survivors: Results of the first randomized single-center controlled trial. Gynecol. Oncol..

[22] Stearns V., Slack R., Greep N., Henry-Tilman R., Osborne M., Bunnell C., Ullmer L., Gallagher A., Cullen J., Gehan E. (2005). Paroxetine is an effective treatment for hot flashes: Results from a prospective randomized clinical trial. J. Clin. Oncol..

[23] Pinkerton J.V., Joffe H., Kazempour K., Mekonnen H., Bhaskar S., Lippman J. (2015). Low-dose paroxetine (7.5 mg) improves sleep in women with vasomotor symptoms associated with menopause. Menopause.

[24] Weitzner M.A., Moncello J., Jacobsen P.B., Minton S. (2002). A pilot trial of paroxetine for the treatment of hot flashes and associated symptoms in women with breast cancer. J. Pain Symptom Manag..

[25] Higgins J.P.T., Green S. (2011). Cochrane Handbook for Systematic Reviews of Intervention.

[26] Mirkin S., Archer D.F., Pickar J.H., Komm B.S. (2015). Recent advances help understand and improve the safety of menopausal therapies. Menopause.

[27] Carpenter J., Gass M.L., Maki P.M., Newton K.M., Pinkerton J.V., Taylor M., Shifren J.L. (2015). Nonhormonal management of menopause-associated vasomotor symptoms: 2015 position statement of The North American Menopause Society. Menopause.

[28] Laganà A.S., Condemi I., Retto G., Muscatello M.R., Bruno A., Zoccali R.A., Triolo O., Cedro C. (2015). Analysis of psychopathological comorbidity behind the common symptoms and signs of endometriosis. Eur. J. Obstet. Gynecol. Reprod. Biol..

[29] Vitale S.G., Laganà A.S., Muscatello M.R., La Rosa V.L., Currò V., Pandolfo G., Zoccali R.A., Bruno A. (2016). Psychopharmacotherapy in Pregnancy and Breastfeeding. Obstet. GynecolSurv..

[30] Rizzo M.R., Leo S., De Franciscis P., Colacurci N., Paolisso G. (2014). Short-term effects of low-dose estrogen/drospirenone vs low-dose estrogen/dydrogesterone on glycemic fluctuations in postmenopausal women with metabolic syndrome. Age.

[31] Slaton R.M., Champion M.N., Palmore K.B. (2015). A review of paroxetine for the treatment of vasomotor symptoms. J. Pharm. Pract..

[32] Wei D., Chen Y., Wu C., Wu Q., Yao L., Wang Q., Wang X.Q., Yang K.H. (2016). Effect and safety of paroxetine for vasomotor symptoms: Systematic review and meta-analysis. BJOG Int. J. Obstet. Gynaecol..

